# Redetermination of 3-deaza­uracil

**DOI:** 10.1107/S1600536808014578

**Published:** 2008-05-17

**Authors:** Gustavo Portalone

**Affiliations:** a Chemistry Department, "Sapienza" University of Rome, P.le A. Moro, 5, I-00185 Rome, Italy

## Abstract

The crystal structure of the title compound, 4-hydr­oxy-2-pyridone, C_5_H_5_NO_2_, which has been the subject of several determinations using X-rays and neutron diffraction, was first reported by Low & Wilson [*Acta Cryst*. (1983). C**39**, 1688–1690]. It has been redetermined, providing a significant increase in the precision of the derived geometric parameters. The asymmetric unit comprises a planar 4-enol tautomer having some degree of delocalization of π-electron density through the mol­ecule. In the crystal structure, the mol­ecules are connected into chains by two strong O—H⋯O and N—H⋯O hydrogen bonds between the OH and NH groups and the carbonyl O atom.

## Related literature

For previous structure determinations, see: Low & Wilson (1983 ▶); Wilson *et al.* (1992 ▶); Wilson (1994 ▶, 2001 ▶). For related literature, see: Stewart & Jensen (1967 ▶): Robins *et al.* (1969 ▶); Schwalbe & Saenger (1973 ▶). For a general approach to the use of multiple-hydrogen-bonding DNA/RNA nucleobases as potential supra­molecular reagents, see: Portalone *et al.* (1999 ▶); Portalone & Colapietro (2004 ▶, 2007 ▶ and references therein). For high-order refinement, see: Hirshfeld (1992 ▶). For the computation of ring patterns formed by hydrogen bonds in crystal structures, see: Etter *et al.* (1990 ▶); Bernstein *et al.* (1995 ▶); Motherwell *et al.* (1999 ▶).
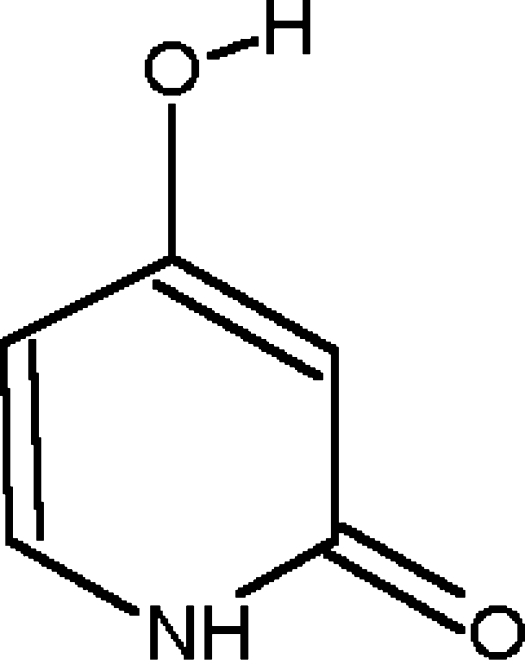


## Experimental

### 

#### Crystal data

C_5_H_5_NO_2_
*M* *_r_* = 111.10
Orthorhombic, 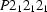

*a* = 5.3393 (1) Å
*b* = 8.6454 (1) Å
*c* = 11.2652 (2) Å
*V* = 520.01 (1) Å^3^
*Z* = 4
Mo *K*α radiation
μ = 0.11 mm^−1^
*T* = 298 (2) K
0.20 × 0.15 × 0.15 mm

#### Data collection

Oxford Diffraction Xcalibur S CCD diffractometer
Absorption correction: multi-scan (*CrysAlis RED*; Oxford Diffraction, 2006 ▶) *T*_min_ = 0.924, *T*_max_ = 0.983
77963 measured reflections
1085 independent reflections
1043 reflections with *I* > 2σ(*I*)
*R*_int_ = 0.025

#### Refinement

*R*[*F* ^2^ > 2σ(*F* ^2^)] = 0.038
*wR*(*F* ^2^) = 0.112
*S* = 1.10
1085 reflections
93 parameters
All H-atom parameters refined
Δρ_max_ = 0.22 e Å^−3^
Δρ_min_ = −0.13 e Å^−3^

### 

Data collection: *CrysAlis CCD* (Oxford Diffraction, 2006 ▶); cell refinement: *CrysAlis RED* (Oxford Diffraction, 2006 ▶); data reduction: *CrysAlis RED*; program(s) used to solve structure: *SIR97* (Altomare *et al.*, 1999 ▶); program(s) used to refine structure: *SHELXL97* (Sheldrick, 2008 ▶); molecular graphics: *ORTEP-3* (Farrugia, 1997 ▶); software used to prepare material for publication: *WinGX* (Farrugia, 1999 ▶).

## Supplementary Material

Crystal structure: contains datablocks global, I. DOI: 10.1107/S1600536808014578/kp2171sup1.cif

Structure factors: contains datablocks I. DOI: 10.1107/S1600536808014578/kp2171Isup2.hkl

Additional supplementary materials:  crystallographic information; 3D view; checkCIF report

## Figures and Tables

**Table 1:** Hydrogen-bond geometry (Å, °)

*D*—H⋯*A*	*D*—H	H⋯*A*	*D*⋯*A*	*D*—H⋯*A*
O2—H2⋯O1^i^	0.97 (2)	1.62 (2)	2.5886 (16)	171.3 (16)
N1—H1⋯O1^ii^	0.89 (2)	1.94 (2)	2.8024 (14)	160.6 (15)

Symmetry codes: (i) 
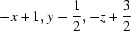
; (ii) 
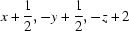
.

**Fig. 1.**
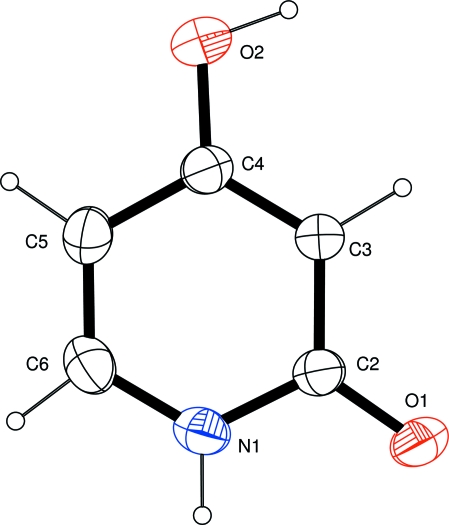
The molecular structure of (I), showing the atom-labelling scheme. Displacements ellipsoids are at the 50% probability level.

**Fig. 2.**
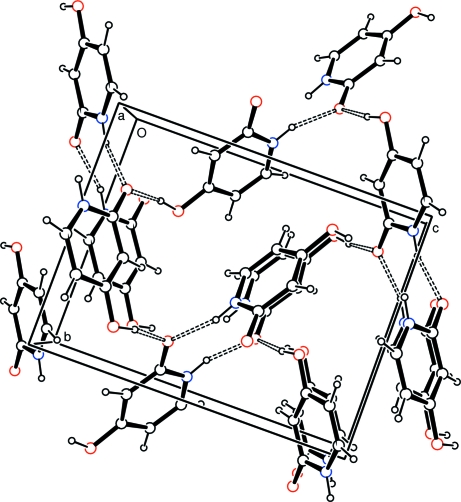
Crystal packing diagram for (I) viewed approximately down a. All atoms are shown as small spheres of arbitrary radii. Hydrogen bonding is indicated by dashed lines.

## supplementary crystallographic information

### Comment

The crystal structure of the modified nucleic acid base 3-deazauracil, 3deazur, has been the subject of several structural studies using XD and ND techniques in order to localise the H atoms as accurately as possible and to define their functions;  the very strong hydrogen bond is found in the structure, as also observed in the parent nucleoside (3-deazauridine) (Schwalbe & Saenger, 1973). This interaction might play a relevant role in the powerful cytostatic properties of the nucleoside (Robins *et al.*, 1969). In the first crystal structure determination (XRD, Low & Wilson, 1983) 713 unique reflections were collected at ambient temperature on an automatic diffractometer. H atoms were localized by a difference Fourier map with the exception of the hydroxyl H2, and all but H1, which was fixed at its position as obtained from the difference map, were included as riding atoms at calculated positions. The final refinement led to *R* = 0.065, and standard deviations of 0.004Å in C—C bond lengths and 0.3° in bond angles. Subsequently, a joint X-ray and neutron diffraction blocked-matrix refinement, based on limited neutron single-crystal data (80) combined with 674 X-ray data, was reported (*X*—N, Wilson *et al.*, 1992). This calculation, involving 94 refined parameters with all but the H atoms treated anisotropically, led to *R* = 0.075. Two years later, in a pulsed neutron single-crystal study (PND, Wilson, 1994), 119 parameters and 447 unique reflections [with I > 5σ(I)] yielded an *R* = 0.071, with a poor agreement between the geometrical parameters obtained from this and the previous experiments (*e.g.* the N1–C2 bond distance is 1.332 (6) in PND, 1.362 (6) in *X*—N and 1.360 (4) in XRD, Table 2). Finally, in a neutron single-crystal diffraction experiment at 100 K using a sample containing one crystal of 3-deazauracil and one of lead hydrogen arsenate (LTND, Wilson, 2001). 118 parameters and 1426 unique reflections [with I > 2σ(I)] yielded an *R* = 0.078. Again, some discrepancies between the geometrical parameters from this study and those from the previous structural determinations remain unsolved (*e.g.* the C4–O2 bond distance is 1.322 (4) in LTND, 1.345 (8) in PND, 1.346 (5) in *X*—N and 1.319 (3) in XRD, Table 2). and it was suggested the presence of tautomeric mixing.

As a part of a more general study of multiple-hydrogen-bonding DNA/RNA nucleobases as potential supramolecular reagents (Portalone *et al.*, 1999; Portalone & Colapietro, 2004, 2007), and in view of the importance of the title compound, this paper reports a redetermination of the crystal structure with greater precision and accuracy.

The asymmetric unit of (I) comprises a planar independent molecule as 4-enol tautomer (Fig. 1). A comparison of the molecular geometry of 3-deazur (high θ refinement) with that reported for uracil (Stewart & Jensen, 1967), in conjunction with a detailed examination of Fourier maps, exclude the presence of tautomeric mixing (4-enol and 2-enol tautomers) and suggests some degree of delocalization of π-electron density through the 4-enol tautomer, which in turn strengthens the existing intermolecular hydrogen bonds (Portalone *et al.*, 1999). Analysis of the crystal packing of (I) shows (Table 1) that the structure is stabilized by two strong independent intermolecular O—H···O and N—H···O interactions of descriptor C^1^_1_(3) (Etter *et al.*, 1990; Bernstein *et al.*, 1995; Motherwell *et al.*, 1999) between OH and NH moieties and the carbonyl O atom (O1^i^ and O1^ii^) [symmetry code: (i) -*x* + 1, *y* - 1/2, -*z* + 3/2; (ii) *x* + 1/2, -*y* + 1/2, -*z* + 2] which link the molecules into chains (Fig. 2).

### Experimental

3-deazauracil (0.1 mmol, Sigma Aldrich at 97% purity) was dissolved in water (9 mL) and heated under reflux for 3 h. After cooling the solution to an ambient temperature, crystals suitable for single-crystal X-ray diffraction were grown by slow evaporation of a solvent after a few days.

### Refinement

All H atoms were found in a difference map and refined isotropically. In the high θ refinement (Hirshfeld, 1992) 624 reflections having H_min_ = 0.9 Å^-1^ and 74 parameters yielded the following results: R1 = 0.0339, wR2 = 0.0679, S = 1.190. H atoms were kept fixed at values deduced from the conventional refinement. In the absence of significant anomalous scattering in this light-atom study, Friedel pairs were merged.

### Crystal data

**Table:**

C_5_H_5_NO_2_	*D*_x_ = 1.419 Mg m^−^^3^
*M**_r_* = 111.10	Melting point: 477 K
Orthorhombic, *P*2_1_2_1_2_1_	Mo *K*α radiation λ = 0.71073 Å
Hall symbol: P 2ac 2ab	Cell parameters from 43477 reflections
*a* = 5.3393 (1) Å	θ = 2.9–32.3º
*b* = 8.6454 (1) Å	µ = 0.11 mm^−^^1^
*c* = 11.2652 (2) Å	*T* = 298 (2) K
*V* = 520.006 (14) Å^3^	Tablets, colourless
*Z* = 4	0.20 × 0.15 × 0.15 mm
*F*_000_ = 232	

### Data collection

**Table:**

Oxford Diffraction Xcalibur S CCD diffractometer	1085 independent reflections
Radiation source: Enhance (Mo) X-ray source	1043 reflections with *I* > 2σ(*I*)
Monochromator: graphite	*R*_int_ = 0.025
Detector resolution: 16.0696 pixels mm^-1^	θ_max_ = 32.4º
*T* = 298(2) K	θ_min_ = 3.0º
ω and φ scans	*h* = −8→7
Absorption correction: multi-scan(CrysAlis RED; Oxford Diffraction, 2006)	*k* = −13→12
*T*_min_ = 0.924, *T*_max_ = 0.983	*l* = −16→16
77963 measured reflections	

### Refinement

**Table:**

Refinement on *F*^2^	Secondary atom site location: difference Fourier map
Least-squares matrix: full	Hydrogen site location: inferred from neighbouring sites
*R*[*F*^2^ > 2σ(*F*^2^)] = 0.038	All H-atom parameters refined
*wR*(*F*^2^) = 0.112	*w* = 1/[σ^2^(*F*_o_^2^) + (0.0765*P*)^2^ + 0.037*P*] where *P* = (*F*_o_^2^ + 2*F*_c_^2^)/3
*S* = 1.10	(Δ/σ)_max_ < 0.001
1085 reflections	Δρ_max_ = 0.22 e Å^−^^3^
93 parameters	Δρ_min_ = −0.13 e Å^−^^3^
Primary atom site location: structure-invariant direct methods	Extinction correction: none

### Special details

**Table:**

Experimental. (CrysAlis RED; Oxford Diffraction Ltd., Version 1.171.31.7 (release 18-10-2006 CrysAlis171 .NET) (compiled Oct 18 2006,16:28:17) Empirical absorption correction using spherical harmonics, implemented in SCALE3 ABSPACK scaling algorithm.
Geometry. All e.s.d.'s (except the e.s.d. in the dihedral angle between two l.s. planes) are estimated using the full covariance matrix. The cell e.s.d.'s are taken into account individually in the estimation of e.s.d.'s in distances, angles and torsion angles; correlations between e.s.d.'s in cell parameters are only used when they are defined by crystal symmetry. An approximate (isotropic) treatment of cell e.s.d.'s is used for estimating e.s.d.'s involving l.s. planes.
Refinement. Refinement of *F*^2^ against ALL reflections. The weighted *R*-factor *wR* and goodness of fit *S* are based on *F*^2^, conventional *R*-factors *R* are based on *F*, with *F* set to zero for negative *F*^2^. The threshold expression of *F*^2^ > σ(*F*^2^) is used only for calculating *R*-factors(gt) *etc*. and is not relevant to the choice of reflections for refinement. *R*-factors based on *F*^2^ are statistically about twice as large as those based on *F*, and *R*- factors based on ALL data will be even larger.

### Fractional atomic coordinates and isotropic or equivalent isotropic displacement parameters (Å^2^)

**Table:**

	*x*	*y*	*z*	*U*_iso_*/*U*_eq_	
O1	0.5630 (2)	0.19169 (12)	0.88298 (9)	0.0418 (3)	
O2	0.8080 (2)	−0.31430 (11)	0.76490 (11)	0.0423 (3)	
H2	0.667 (4)	−0.302 (3)	0.711 (2)	0.056 (6)*	
N1	0.9062 (2)	0.07483 (13)	0.96127 (9)	0.0329 (2)	
H1	0.935 (5)	0.163 (3)	1.000 (2)	0.052 (6)*	
C2	0.7058 (2)	0.07410 (13)	0.88575 (10)	0.0289 (2)	
C3	0.6712 (2)	−0.06036 (13)	0.81646 (10)	0.0287 (2)	
H3	0.523 (4)	−0.072 (2)	0.763 (2)	0.056 (6)*	
C4	0.8348 (2)	−0.18253 (14)	0.82563 (11)	0.0299 (3)	
C5	1.0428 (3)	−0.17405 (17)	0.90371 (13)	0.0363 (3)	
H5	1.161 (4)	−0.263 (3)	0.9032 (17)	0.054 (6)*	
C6	1.0714 (3)	−0.04424 (17)	0.96924 (12)	0.0362 (3)	
H6	1.212 (4)	−0.021 (2)	1.0225 (18)	0.045 (5)*	

### Atomic displacement parameters (Å^2^)

**Table:**

	*U*^11^	*U*^22^	*U*^33^	*U*^12^	*U*^13^	*U*^23^
O1	0.0522 (6)	0.0310 (4)	0.0421 (5)	0.0126 (5)	−0.0071 (5)	−0.0089 (4)
O2	0.0473 (6)	0.0304 (4)	0.0493 (5)	0.0056 (5)	−0.0048 (5)	−0.0116 (4)
N1	0.0378 (5)	0.0301 (5)	0.0308 (5)	−0.0046 (4)	−0.0035 (4)	−0.0040 (4)
C2	0.0330 (5)	0.0261 (5)	0.0277 (5)	0.0007 (5)	0.0004 (4)	−0.0020 (4)
C3	0.0291 (5)	0.0270 (5)	0.0301 (5)	0.0014 (4)	−0.0025 (4)	−0.0041 (4)
C4	0.0307 (6)	0.0269 (5)	0.0321 (5)	0.0003 (5)	0.0013 (4)	−0.0020 (4)
C5	0.0306 (6)	0.0352 (6)	0.0432 (6)	0.0044 (5)	−0.0041 (5)	0.0007 (5)
C6	0.0318 (6)	0.0400 (6)	0.0369 (6)	−0.0037 (5)	−0.0070 (5)	0.0015 (5)

### Geometric parameters (Å, °)

**Table:**

O1—C2	1.2713 (15)	C3—C4	1.3746 (16)
O2—C4	1.3366 (15)	C3—H3	1.00 (2)
O2—H2	0.97 (2)	C4—C5	1.4187 (19)
N1—C6	1.3587 (17)	C5—C6	1.3519 (19)
N1—C2	1.3668 (17)	C5—H5	1.00 (2)
N1—H1	0.89 (2)	C6—H6	0.98 (2)
C2—C3	1.4123 (15)		
			
C4—O2—H2	107.8 (14)	O2—C4—C3	123.25 (11)
C6—N1—C2	123.08 (10)	O2—C4—C5	116.44 (11)
C6—N1—H1	120.3 (16)	C3—C4—C5	120.30 (11)
C2—N1—H1	116.2 (16)	C6—C5—C4	118.02 (12)
O1—C2—N1	118.75 (10)	C6—C5—H5	125.0 (13)
O1—C2—C3	124.48 (12)	C4—C5—H5	117.0 (13)
N1—C2—C3	116.76 (11)	C5—C6—N1	121.30 (12)
C4—C3—C2	120.51 (11)	C5—C6—H6	126.0 (12)
C4—C3—H3	118.3 (12)	N1—C6—H6	112.6 (12)
C2—C3—H3	121.1 (12)		

### Hydrogen-bond geometry (Å, °)

**Table:**

*D*—H···*A*	*D*—H	H···*A*	*D*···*A*	*D*—H···*A*
O2—H2···O1^i^	0.97 (2)	1.62 (2)	2.5886 (16)	171.3 (16)
N1—H1···O1^ii^	0.89 (2)	1.94 (2)	2.8024 (14)	160.6 (15)

Symmetry codes: (i) −*x*+1, *y*−1/2, −*z*+3/2; (ii) *x*+1/2, −*y*+1/2, −*z*+2.

### Table 2 Comparison of selected interatomic distances and angles (Å,°) for the title compound and uracil

**Table:**

	3Deazur						Uracil
	This work^a^	This work^b^	LTND^c^	PND^d^	X-N^e^	XRD^f^	XRD^g^
N1-C2	1.3668 (17)	1.3696 (13)	1.362 (3)	1.332 (6)	1.362 (6)	1.360 (4)	1.371 (3)
N1-C6	1.3587 (17)	1.3577 (15)	1.355 (3)	1.356 (6)	1.367 (7)	1.359 (4)	1.358 (2)
C2-C3	1.4123 (15)	1.4141 (11)	1.414 (3)	1.409 (7)	1.409 (6)	1.412 (4)	—
C5-C6	1.3519 (19)	1.3587 (15)	1.360 (4)	1.350 (8)	1.355 (7)	1.348 (4)	1.340 (2)
C3-C4	1.3746 (16)	1.3860 (12)	1.385 (4)	1.381 (7)	1.375 (7)	1.381 (4)	—
C4-C5	1.4187 (19)	1.4183 (15)	1.411 (4)	1.417 (8)	1.400 (7)	1.411 (4)	1.430 (3)
C2-O1	1.2713 (15)	1.2735 (12)	1.266 (4)	1.268 (7)	1.266 (6)	1.262 (4)	1.215 (2)
C4-O2	1.3366 (15)	1.3342 (11)	1.322 (4)	1.345 (8)	1.346 (5)	1.319 (3)	1.245 (2)
O2···O1^i^	2.5886 (16)	2.5843 (16)	2.563 (4)	2.575 (11)	2.581 (6)	2.550 (4)	—
N1···O1^ii^	2.8024 (14)	2.7998 (12)	2.785 (3)	2.776 (8)	2.796 (5)	2.807 (4)	2.861 (2)
O2-H2···O1^i^	171.3 (16)	172.8 (9)	177.7 (7)	174.6 (13)	166 (4)	—	—
N1-H1···O1^ii^	160.6 (15)	164.7 (8)	165.5 (6)	164.0 (10)	158 (4)	156	171 (1)

Notes: (a) `Conventional' refinement; (b) High θ refinement; (c) Low Temperature ND (Wilson, 2001); (d) Pulsed ND (Wilson, 1994); (e) Joint X-N (Wilson *et al.*,  1992); (f) XRD (Low & Wilson, 1983); (g) XRD (Stewart & Jensen, 1967). Symmetry codes: (i) -x+1, y-1/2, -z+3/2; (ii) x+1/2, -y+1/2, -z+2.

## References

[bb1] Altomare, A., Burla, M. C., Camalli, M., Cascarano, G. L., Giacovazzo, C., Guagliardi, A., Moliterni, A. G. G., Polidori, G. & Spagna, R. (1999). *J. Appl. Cryst.* **32**, 115–119.

[bb2] Bernstein, J., Davis, R. E., Shimoni, L. & Chang, N.-L. (1995). *Angew. Chem. Int. Ed. Engl.* **34**, 1555–1573.

[bb3] Etter, M. C., MacDonald, J. C. & Bernstein, J. (1990). *Acta Cryst.* B**46**, 256–262. 10.1107/s0108768189012929 2344397

[bb4] Farrugia, L. J. (1997). *J. Appl. Cryst.* **30**, 565.

[bb5] Farrugia, L. J. (1999). *J. Appl. Cryst.* **32**, 837–838.

[bb6] Hirshfeld, F. L. (1992). *Accurate Molecular Structures. Their Determination and Importance*, edited by A. Domenicano & I. Hargittai, pp 252–253. New York: Oxford University Press.

[bb7] Low, J. N. & Wilson, C. C. (1983). *Acta Cryst.* C**39**, 1688–1690.

[bb8] Motherwell, W. D. S., Shields, G. P. & Allen, F. H. (1999). *Acta Cryst.* B**55**, 1044–1056. 10.1107/s0108768199006497 10927446

[bb9] Oxford Diffraction (2006). *CrysAlis CCD* and *CrysAlis RED* Oxford Diffraction Ltd, Abingdon, Oxfordshire, England.

[bb10] Portalone, G., Bencivenni, L., Colapietro, M., Pieretti, A. & Ramondo, F. (1999). *Acta Chem. Scand.* **53**, 57–68.

[bb11] Portalone, G. & Colapietro, M. (2004). *Acta Cryst.* E**60**, o1165–o1166.

[bb12] Portalone, G. & Colapietro, M. (2007). *Acta Cryst.* C**63**, o181–o184. 10.1107/S0108270107005483 17339726

[bb13] Robins, M. J., Currie, B. L., Robins, L. K. & Bloch, A. (1969). *Proc. Am. Assoc. Cancer Res.* **10**, 73–79.

[bb14] Schwalbe, C. H. & Saenger, W. (1973). *Acta Cryst.* B**29**, 61–69.

[bb15] Sheldrick, G. M. (2008). *Acta Cryst.* A**64**, 112–122. 10.1107/S0108767307043930 18156677

[bb16] Stewart, R. F. & Jensen, L. H. (1967). *Acta Cryst.* **23**, 1102–1105.

[bb17] Wilson, C. C. (1994). *J. Chem. Crystallogr.* **24**, 371–373.

[bb18] Wilson, C. C. (2001). *J. Mol. Struct.* **560**, 239–246.

[bb19] Wilson, C. C., Keen, D. & Stewart, N. S. (1992). *J. Chem. Soc. Chem. Commun.* pp. 1160–1162.

